# Entrance Requirements to German Dental Schools

**Published:** 1902-11-15

**Authors:** 


### ENTRANCE REQUIREMENTS TO GERMAN DENTAL SCHOOLS.

The Society of Professors of Dentistry of the German Universities, which is presided over by Dr. Miller, of Berlin, has again addressed a petition to the Federal Council, the text of which is as follows: "That the candidates for the diploma of dental surgeon shall not be permitted to take the examinations if they are not holders of a certificate of *maturite* (a German certificate of *maturite* is equivalent to an American baccalaureate degree) ; and that the permission to substitute two semesters of study at the university by an apprenticeship of one year at a dentist's office shall be suppressed, and that all the studies which are part of the dental curriculum shall be pursued at the university.'
—*Cosmos*.

